# Physico- and phytochemical properties of *Brassica juncea* as affected by agroclimatic conditions

**DOI:** 10.1038/s41598-023-48808-9

**Published:** 2024-01-08

**Authors:** Uzma Batool, Rab Nawaz, Sajjad Ahmad, Muhammad Atif Irshad, Ali Irfan, Abdel-Rhman Z. Gaafar, Muhammad Arshad, Gezahign Fentahun Wondmie, Mir Muhammad Nasir Qayyum, Mohammed Bourhia

**Affiliations:** 1 State Key Laboratory of Food Science and Technology, Jiangnan University, Wuxi, Jiangsu, 214122, China; 2 Department of Environmental Sciences, The University of Lahore, Lahore, 54000 Pakistan; 3 Faculty of Engineering and Quantity Surveying, INTI International University, 71800 Nilai, Negeri Sembilan, Malaysia; 4 Department of Civil Engineering, COMSATS University Islamabad, Sahiwal Campus, Sahiwal, 57000, Pakistan; 5 Department of Chemistry, Government College University Faisalabad, Faisalabad, 38000 Pakistan; 6 Department of Botany and Microbiology, College of Science, King Saud University, P.O. Box 11451, Riyadh, Saudi Arabia; 7 Department of Agriculture & Food Technology, Karakoram International University, Gilgit, 15100 Pakistan; 8 Department of Biology, Bahir Dar University, P.O. Box 79, Bahir Dar, Ethiopia; 9 Department of Chemistry and Biochemistry, Faculty of Medicine and Pharmacy, Ibn Zohr University, Laayoune, Morocco; 10 Laboratory of Chemistry-Biochemistry, Environment, Nutrition, and Health, Faculty of Medicine and Pharmacy, University Hassan II, B. P. 5696 Casablanca, Morocco

**Keywords:** Plant sciences, Ecology

## Abstract

Physicochemical and phytochemical assessment of leaf mustard (*Brassica juncea *L.) grown in different agroclimatic conditions is essential to highlight their compositional variability and evaluate the most suitable bunch of agroclimatic and agronomic practices. *B. juncea* is one of the important leafy vegetables that serve as source of vitamin A and C and iron, and plenty of antioxidants. This in situ research was executed to assess the quality variability of *B. juncea* grown in different agroecosystems. Leaves’ samples of *B. juncea* were procured from 15 farmers’ fields exhibiting different agroclimatic conditions i.e., elevation, nutrient management, temperature, irrigation, and tillage practices. Leaves’ samples were subjected to physicochemical and phytochemical analysis, i.e., moisture, pH, TSS, ascorbic acid, carotenoids, phenolics, flavonoids, and antioxidant potential. In the leaves’ samples of *B. juncea*, the target properties were found to vary significantly (*P* ≤ 0.05) in different agroclimatic conditions. The moisture content, ascorbic acid, phenolic content, carotenoids, and antioxidants were found in the range of 62.7–79.3%, 74–91 mg/100 g, 49.2–49.2 mg GAE/100 g, 436.3–480 mg β carotene/100 g, 32.7–46.67%, respectively. This study elaborates the significant variation of physicochemical and phytochemical attributes of *B. juncea* due to the prevailing agroclimatic conditions. This necessitates the appropriate choice of *B. juncea* concerning its composition and ecological conditions of its cultivation in the prospective health benefits.

## Introduction

Leaf Mustard (*Brassica juncea* L.) contains countless health-supporting plant chemicals including glucosinolates, carotenoids, and phenolic compounds^1^. These complexes are frequently linked with their capability to turn as detoxifiers counter to oxidative anxiety^2^. This vegetable also called Chinese mustard, Indian mustard, mustard green, oriental mustard, and leaf mustard is a species of family Brassicaceae or Mustard family^3^. These Brassica plant species are well known for their antioxidant properties associated with health benefits for the human body^4^. It originated from Central Asia and then spread to Pakistan, Western and Central China, Eastern India, Iran, Burma, Central Africa, Japan, and Nepal and now also grown in Southern Russia towards the North of the Caspian Sea^5^.

It contains dietary fibers, vitamins, minerals and flavonoids, and has an inherent ability to inhibit or cure diabetes through its antioxidant potential^6^. It contains many bio-chemicals, i.e., carbohydrates, proteins, phenols, sterols, glycosides, and flavonoids^7^. Leaf mustard contains ascorbic acid, flavonoids and polyphenols which are phytochemicals and play important role as antioxidants because of their reducing capability and hydrogen donating properties^8^. Polyphenols are hydroxycinnamic acids and flavonoids which are abundantly found in brassica plants^9^. *Brassica juncea* as a meal is a good and compact source of phenolic compounds but these compounds were not desirable in ancient times as they produce a bitter and astringent flavour and dark colour^10^. But now these compounds got attention due to their antioxidant properties for value addition to different foods^11^.

Brassica stems, leaves, and seeds are beneficial for reducing high blood pressure problems, migraine issues, diabetes, and severe asthma^12^. It is also helpful in normalizing sleeping issues, preventing coronary heart problems, and delaying women facing menopausal pattern issues^13^. It has various uses and is economically an important plant being a source of oil and a green leafy vegetable accompanied by various medicinal properties^14^. Its consumption can provide lots of vitamins and micronutrients such as e, β-carotenoids, vitamin C, and many antioxidants. It is known for its eco-friendly nature as it is helpful in the prevention of many non-communicable diseases due to its nutraceutical and drug properties^15^. Antioxidants are comprised of various collections of compounds and these antioxidant enzymes are manufactured naturally in the human body and are also found in plant materials which help us in rep-oxidant-induced injury in our body^16^. Similarly, phytochemicals are non-nutritive compounds extracted from plants that help in defending the human body from many harmful diseases. These prevent oxidation and remove harmful free radicals from the body^17^. Green leafy vegetables play a significant role in a balanced diet because of many phytochemicals and nutritional components^18^.

Agroclimatic conditions are comprised of cultivation practices, nutrient management, irrigation, and long-term weather conditions along the elevation gradient. These conditions have a significant influence on the quantity and quality of agricultural production^19,20^. Effect of drought conditions has been noted on mustard yield and grain quality by other study^21^. Air temperature variation and ozone concentration significantly influence the metabolism and yield of *Brassica juncea*^22,23^.

Leaf mustard is the most eaten green leafy vegetable as fresh as well as in dried form. Plants from the Brassica family are enriched in phytochemicals^4^. Many plants of mustard family have been studied by different researchers for phytochemical and antioxidant profile while sampling from a single agroclimatic condition at a time^1,24^. Effect of different agroclimatic conditions on the physico- and phytochemical properties of *Brassica juncea* has not been addressed in previous studies. Therefore, purpose of the present research study was to investigate and assess the variability of physico and phytochemical constituents of *Brassica juncea* and to evaluate the most appropriate agroclimatic conditions for the successful cultivation of healthful *Brassica juncea*.

## Results and discussion

### Physicochemical properties

**Figure 1:**
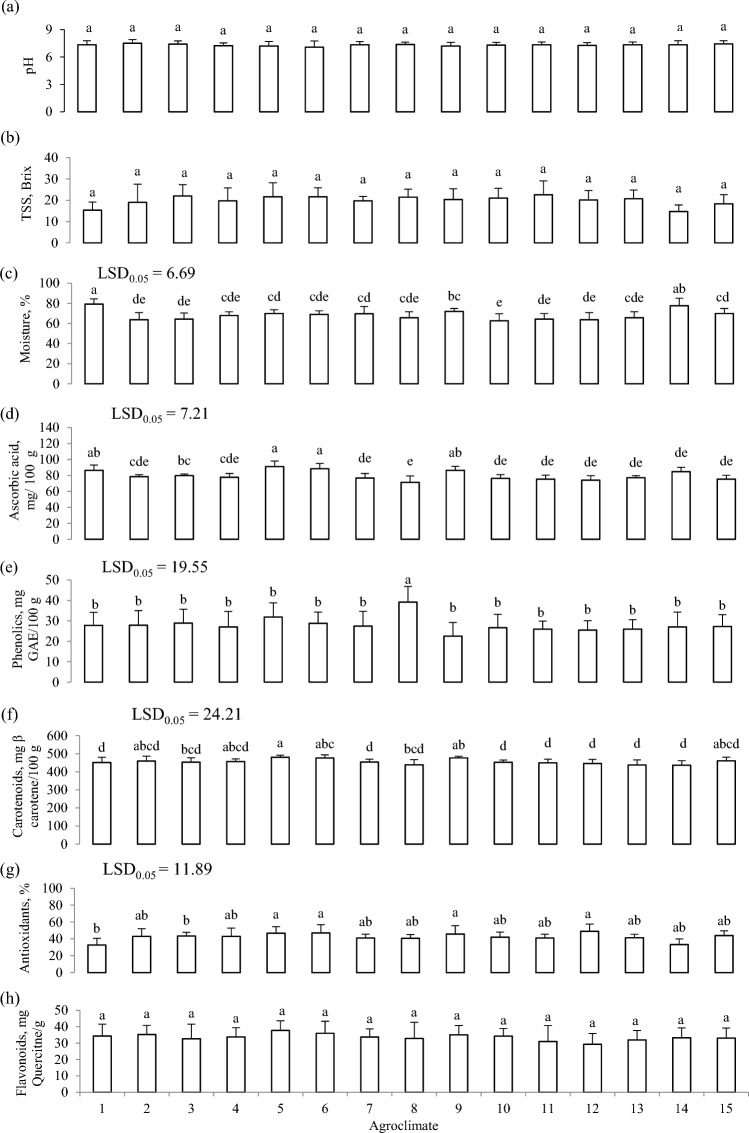
Physicochemical attributes: pH (**a**), total soluble solids (**b**), moisture (**c**), ascorbic acid (**d**), phenolics (**e**), carotenoids (**f**), antioxidants (**g**), and flavonoids (**h**) of leaf mustard samples from 15 different agroclimatic conditions.

The pH of leaf sap was in the range of 6.3–7.9 with a mean value of 7.3 (Fig. 1a). It was the highest in agroclimatic condition 2 (7.5) and the minimum was in agroclimatic condition 6 (7.1). Statistical analysis showed a non-significant (*P* ≥ 0.05) difference for pH among the samples procured from various agroclimatic conditions. These values are in line with results of previous studies in which pH in *Brassica juncea* leaves was noted as 7.17^25^.

TSS were examined across various samples of *Brassica juncea* collected from diverse agroclimatic conditions, falling within the range of 14.7–22.6 degrees Brix (Fig. 1b). Among the samples, the highest TSS value was found in samples collected from agroclimatic condition 11 which was 22.6° Brix while TSS of 14.7° Brix as the lowest was examined in samples from agroclimatic condition 14. Analysis of data showed a non-significant (*P* ≥ 0.05) difference for TSS among the samples procured from various agroclimatic conditions. These results agree with a study in which TSS in *Brassica juncea* was found as 18%^26^. The TSS value noted in this study was higher than the TSS in *Brassica juncea* observed in another study which was 5.5 mg/g^27^. Different TSS of samples from different areas might be due to topography, soil salinity, fertilization, irrigation, tillage, and agroclimatic conditions.

Moisture content in all samples of *Brassica juncea* from each of the sampling agroclimates was found within the range of 62.67–79.33% (Fig. 1c). The maximum value of 79.33% was found in samples from agroclimatic condition 1. The lowest moisture content 62.67% among the samples was found in leaves collected from agroclimatic conditions 10. Analysis of data indicated a very highly significant (*P* ≤ 0.05) difference on moisture content among the samples procured from various agroclimatic conditions. Variations observed among the samples could be attributed to environmental factors like temperature, irrigation source, light intensity, and soil texture. These factors, along with pre-harvest and intercultural practices, had a notable impact on the moisture content of the leaf mustard.

Ascorbic acid was quantified for all samples of *Brassica juncea* procured from different agroclimatic conditions and this parameter was within a range of 74–91 mg/100 g (Fig. 1d). Amongst all samples it was observed that the maximum value of ascorbic acid was found in samples from agroclimatic conditions 5 and 6 whereas the lowest value which was 74 mg/100g that was recorded for agroclimatic condition 8. Data showed a very highly significant (*P* ≤ 0.05) difference for ascorbic acid among the samples procured from various agroclimatic conditions. Research results are also within the range of findings of ascorbic acid content in *Brassica juncea* and it was found as 88 mg/g^26^. While current results are higher than the results of a study where ascorbic acid in *Brassica juncea* was noted as 0.8 mg/g Differences among samples of ascorbic acid could be due to different agroclimatic conditions and agricultural practices. That’s why it was noted that ascorbic acid is richly found in samples collected from agroclimatic conditions 5 and 6 as these have loamy soil which is helpful for plants to absorb nutrients. From the above study, it can be suggested that locations with high nitrogenised fertilization of plant can have high ascorbic acid in vegetables.

### Phytochemical constituents

Total phenolics content was measured for *Brassica juncea* collected from different locations and it was found that total phenolics were ranged as 22.5–49.17 mg GAE/100 g (Fig. 1e). It was found that the highest phenolic content in mustard samples was 49.17 mg GAE/100 g that was noted in samples from agroclimatic condition 8 (49.2 mg GAE/100 g). Lowest phenolic content was observed in samples from agroclimatic condition 9 and it was 22.5 mg GAE/100 g. In rest of the agroclimatic conditions, phenolic content was within thee limit. Data analysis revealed a noteworthy disparity (*P* ≤ 0.05) in total phenolic content among the samples obtained from different locations. It was observed that these results are in line with the results of a study in which phenolic content ranged from 2.68 to 7.48 mg/g in different cultivars of *Brassica juncea*^2^ and research results are lower than the results previously reported in which it was found that total phenolic content in *Brassica juncea* was 21.98 mg/g. In another study, it was found that phenols in *Brassica juncea* were found at 8 mg/g which is also less than current findings^27^.

*Brassica juncea* samples from different agroclimatic conditions were analysed for carotenoid contents and it was done with the petroleum ether method and was observed in the range of 436.33–480 mg β carotene/100 g (Fig. 1f). It was investigated that the highest total carotenoids were observed in samples procured from agroclimatic condition 5 that was 480 mg β carotene/100 g whereas lowest was observed in samples procured from agroclimatic condition 14 (436.33 mg β carotene/100 g). While the rest of the samples lied within this range. Statistical analysis showed a significant (*P* ≤ 0.05) difference for carotenoids among the samples procured from various locations. Research results are less than results of this study in which carotenoid content ranged from 476 DW to 1114 mg/g in different cultivars of *Brassica juncea*^2^ and whereas research results are greater than results of another study in which carotenoid content in *Brassica juncea* was found as 1.2 mg/g.

Antioxidant potential of Leaf Mustard was also analysed and was found within the range of 32.67–46.67% (Fig. 1g). It was revealed that highest antioxidant potential (46.67%) was found out in samples procured from agroclimatic condition 12 (49.0%) whereas lowest antioxidant potential which was 32.67% and it was observed from agroclimatic condition 1 (32.7%). Analysis of data showed a significant (*P* ≤ 0.05) difference for antioxidants among the samples procured from different agroclimatic conditions. Current findings agree with study where they found the antioxidant potential ranged from 21.7 to 41.4% in different cultivars of *Brassica juncea*^2^ whereas in another study we found that antioxidant potential in *Brassica juncea* was 31.2% and it was slightly less than the current findings^26^ (Table 1).

Total flavonoid contents were also analysed for samples procured in various agroclimatic conditions and it was observed among the agroclimatic conditions that total flavonoid contents were in the range of 29.23–37.73 mg Quercitne/g. The highest flavonoid content (37.73 mg Quercitne/g) found was observed in samples procured from agroclimatic condition 5 while lowest flavonoid content among samples was found in agroclimatic condition 12 and it was 29.23 mg Quercitne/g and rest of samples results were within above mentioned range. Analysis of data indicated a non-significant (*P* ≥ 0.05) difference for flavonoids among the samples procured from various locations (Fig. 1h). These findings are in line with results of research where flavonoid content in *Brassica juncea* was found as 31.2 mg/g^25^. The summary of results (Table 2) indicated that higher concentrations of ascorbic acid, carotenoid, and flavonoids were recorded at field sites supplied with cow and goat manures, river irrigation, and manual tillage practices. The highest antioxidants were noted in vegetables grown in a field managed with goat manure as a source of nutrients, river irrigation, and manual tillage operations. Similarly, phenolic content in leaves of *Brassica juncea* were observed in samples collected from plants raised with cow manure, glacier irrigation and manual tillage practices.

**Table 1:**
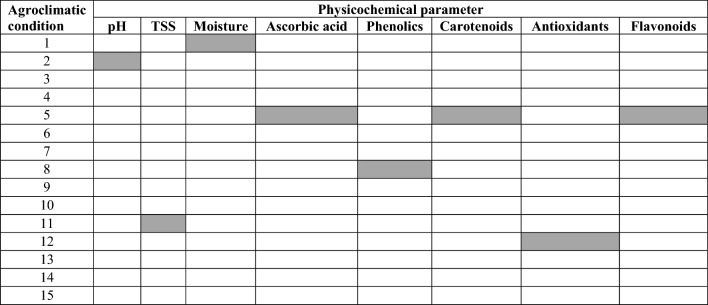
Results summary: maximum values of physicochemical parameters in *Brassica juncea* under different agroclimatic conditions.

### Interrelationship of physicochemical properties

A correlation analysis was conducted to investigate the interplay between the physicochemical and phytochemical characteristics of *Brassica juncea*, revealing a notable association among certain chemical properties (Table 2). There was a significant correlation between pH and total TSS, between moisture content and ascorbic acid, and between ascorbic acid and carotenoids and flavonoids. Similarly, a significant association was observed between carotenoids antioxidants, and flavonoids. Association among physicochemical properties of *Brassica juncea* was also reported by^27^.

**Table 2:** Correlation among physicochemical properties of leaf mustard.

Parameter	pH	TSS	Moisture	Ascorbic acid	Phenolics	Carotenoids
pH	1.00					
TSS	0.39***	1.00				
Moisture	− 0.16^ ns^	− 0.26^ ns^	1.00			
Ascorbic acid	− 0.14^ ns^	− 0.04^ ns^	0.49***	1.00		
Phenolics	0.12^ ns^	0.18^ ns^	0.04^ ns^	− 0.22^ ns^	1.00	
Carotenoids	− 0.20^ ns^	0.10^ ns^	0.26^ ns^	0.53***	0.10^ ns^	1.00
Antioxidants	− 0.23^ ns^	− 0.06^ ns^	− 0.17^ ns^	0.10^ ns^	− 0.22^ ns^	0.53***
Flavonoids	− 0.17^ ns^	0.03^ ns^	0.27^ ns^	0.46***	0.12^ ns^	0.69***

## Conclusions

This research highlighted the variability of physicochemical and phytochemical attributes of *Brassica juncea* which were found significantly varied among different agroclimatic and cultural conditions. Analysis of the tender leaves of *Brassica juncea* procured from different agroclimatic conditions revealed that this vegetable is a good source of natural compounds, especially the antioxidants. Comparison among the leaf samples emphasized that *Brassica juncea* grown in agroclimatic conditions composed of purely organic nutrient management (cow and goat manures), river irrigation, and manual tillage operations is the richest source of ascorbic acid, carotenoids, and flavonoids. However, the highest antioxidant potential was recorded in leaf samples procured from fields where goat manure was used as source of nutrients, river water as irrigation, and manual tillage as cultural practices. Similarly, phenolic content in *Brassica juncea* was the highest where it was grown with river water as irrigation, cow manure as a source of nutrients, and manual tillage as cultivation practice. It was observed that good agronomic practices including favourable nutrient management carried out by the farmers led to an increase in healthy *Brassica juncea* production. Moreover, significant correlation among a few plant parameters indicates their association with each other, that can be managed together and used as potential predictors for yield and physicochemical composition of *Brassica juncea* under different agroclimatic situations.

## Methods

### Agroclimatic conditions of the study area

**Figure 2:**
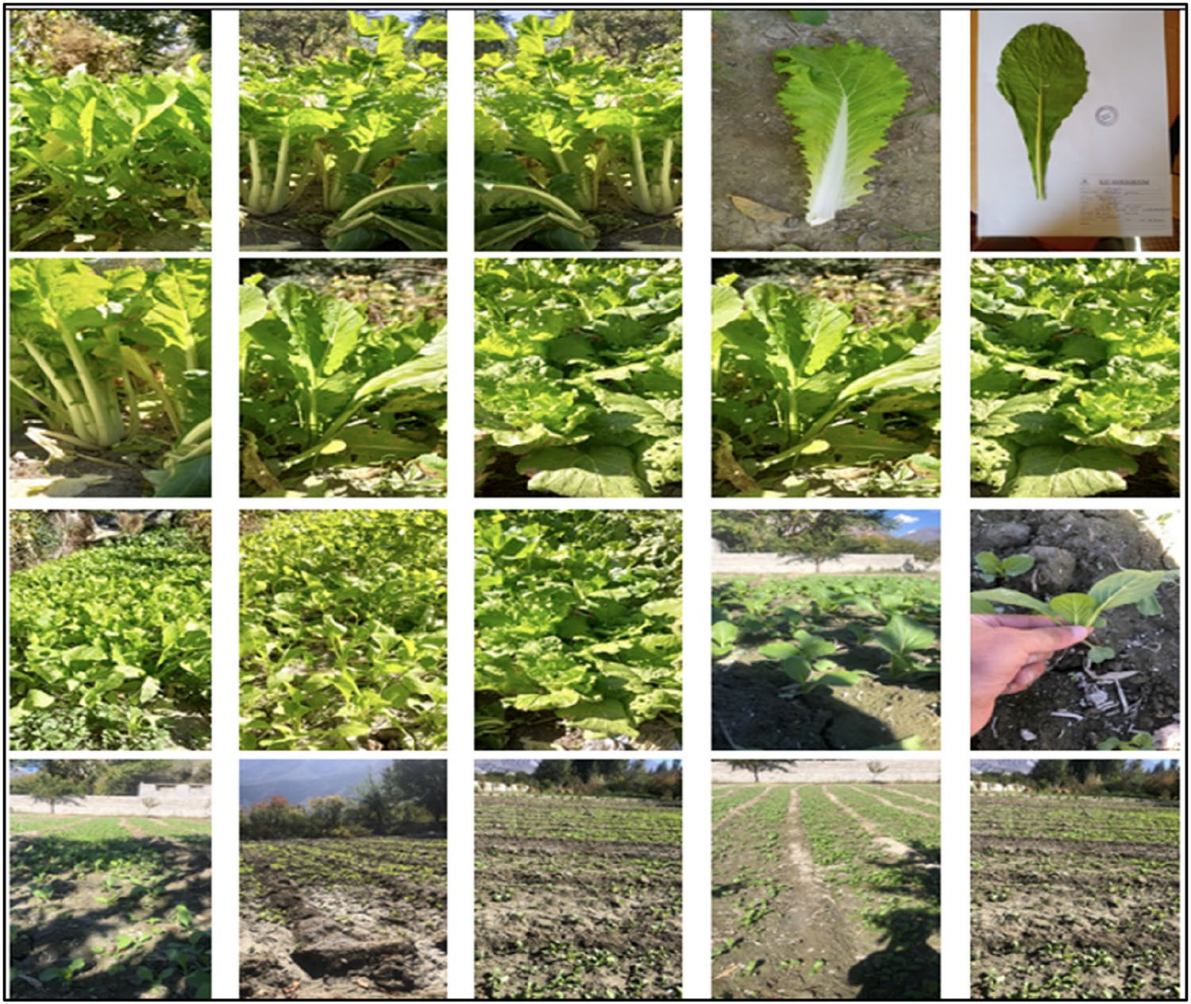
*Brassica juncea* cultivated in different parts of the study area.

The study area was consisted of various farmers’ fields, each distinguished by varying elevations and agroclimatic conditions (Table 3). Farmers of the study area use different cultural, nutrient management, and irrigation practices to grow crops including leafy vegetables at different elevation sites. Tillage operations are carried out using tractors, animals, and manually. Farmers use manures of different animals as nutrient management and irrigate crops through rivers, rainfall, and glacier melts. *Brassica juncea* is one of the leafy vegetables being grown widely the farmers of the study area (Fig. 2).

**Table 3:** Agroclimatic conditions of sampling fields.

Sampling site	Elevation, m	Latitude	Longitude	Temperature range, °C	Agronomic management		
					Source of irrigation	Nutrient management	Tillage
1	2458	36.3591	73.8583	20–29	Glacier + rainfall	CM	Animal
2	2375	36.1857	73.8157	22–32	Glacier	CM	Animal
3	2320	36.1143	74.322	22–30	Glacier + rainfall	GM	Manual
4	2292	36.4552	73.8506	23–33	Glacier	CM	Tractor
5	2281	36.1134	74.3198	23–35	River	CM + GM	Manual
6	2250	36.1553	74.3031	24–35	River	F + CM	Tractor
7	2210	36.3166	74.6512	24–36	Glacier	CM	Tractor
8	2186	36.2991	74.6302	22–36	River	CM	Manual
9	2130	36.4363	73.8416	23–37	Glacier	CM + GM	Manual
10	2125	36.4352	73.8405	24–36	River + rainfall	F + CM	Tractor
11	2066	35.9215	74.4197	25–39	River	F + CM	Manual
12	2052	35.7754	74.5322	28–41	River	GM	Manual
13	2026	35.9070	74.4524	28–39	River + rainfall	F + CM	Tractor
14	2006	35.9080	74.4534	30–38	River	F + GM	Tractor
15	1972	35.9081	74.4535	31–40	River + rainfall	CM + GM	Animal

*CD* cow manure, *GM* goat manure, *F* fertilizer.

### Leaf sample procurement

The present study was conducted to evaluate the physicochemical and phytochemical analysis of cultivated green leafy vegetables called *Brassica juncea* in different agroclimatic zones. Notably, the plant was identified by Dr. Ali Noor Shah, Faculty Member of Department of Plant Sciences, Karakoram International University, Gilgit, Pakistan and a voucher number (NO. KIU-PA-01-3637) was issued before being deposited at the herbarium of the Karakoram International University. The chosen leafy vegetable was subjected to a comparison and evaluation, focusing on its physicochemical properties and antioxidant potential. For this, 15 different agroclimatic conditions were chosen to study the physicochemical and phytochemical characteristics. From each of the agroclimatic conditions, nine (replicates) leaf samples of the vegetable were procured from farmers’ fields. The collected samples were subjected to analysis to examine their physicochemical and phytochemical attributes, encompassing their potential antioxidant properties.

### Sample preparation

Fresh leaves’ samples of vegetables were ground to extract juice for the determination of various parameters except for moisture. For moisture, fresh vegetable samples were dried at 70 °C in an oven. Prepared samples were then analysed for phytochemical and physicochemical constituents.

### Physicochemical analysis

The pH of each sample was calculated by the standard method proposed by Hussain et al*.*^28^ with a digital pH meter (AD 1020). The pH was determined by extracting juice from the leaves of the vegetable to have a liquid matter to dip electrode conveniently. The juice of each sample was taken in 100 mL beakers. Then pH meter was calibrated using buffer solutions of pH 4.0, pH 7.0, and pH 9. Finally, the PH of each sample was measured.

Total soluble solids (TSS) were tested with a digital refractometer using the procedure of Ali and Naz^29^ at ambient temperature. To find TSS vegetable samples were juiced to get liquid composite juice of each vegetable sample was taken in separate beakers to take TSS of each sample. Before each measurement, the lenses of the refractometer were wiped with distilled water.

The percentage of moisture was estimated with the procedure set by Hussain et al.^28^ using Eq. (1). Petri dishes were cleaned and wiped with tissue for drying and were weighed with an electric balance. Vegetable samples were kept in petri dishes for weighing purposes and dishes with samples were kept in an oven for drying at 70 °C. The dishes were then taken out from the oven and cooled in desiccator and this step was repeated several times to achieve a constant weight. Moisture was the computed using formula given in Eq. (1).

1 $$\mathrm{Moisture},\mathrm{ \%}= \frac{\mathrm{Weight\, of\, sample }-\mathrm{weight\, of\, sample\, after\, drying}}{\mathrm{Weight\, of\, sample }}\times 100$$

Ascorbic acid or vitamin C was determined using the standard procedure given by Hussain et al*.*^28^. Firstly, solutions were prepared. The standard solution of dye was made by mixing 42 mg of NaHCO_2_ and 50 mg of 2, 6 dichlorophenol indophenol dyes and was gathered with hot water making volume up to 250 mL. Oxalic acid solution of 0.4% was prepared as well. For making standard ascorbic acid oxalic acid was used to make the volume of 50 mg of ascorbic acid and put in a 50 mL flask. Then vegetable samples were prepared by juicing them from about 100 g of sample. Extracted juice was put in a conical flask for sedimentation. After that 10 mL juice was taken from that sedimented layer put in a beaker and made volume with oxalic acid up to 100 mL. Ten mL of that diluted sample was taken in a conical flask for titration against the dye solution in the burette and titrated till a pink colour was observed in a conical flask. The ally, content of ascorbic acid was found using Eq. (2).

2 $$Ascorbic\, acid= \frac{F\times T\times 100\times 100}{S\times D}$$

where F: mL of ascorbic acid / m of dye used for sample = factor of standardization. T: mL of dye used in the sample. S: mL of sample taken for dilution. D: mL of sample diluted by oxalic acid.

### Phytochemical analysis

Antioxidant inhibition percentage was carried out with the help of the DPPH technique projected by Raza et al*.*^30^ with small changes employed in Eq. (3). A fresh DPPH solution was prepared daily due to the sensitivity of DPPH to heat and light. It was carefully shielded with aluminum foil and stored in a cool location to maintain its stability. To estimate antioxidants in samples, five gram of each sample was first ground for homogenization and then were kept in 10 mL of methanol for 2 days and 0.1 mL was taken out from the homogenized solution in a volumetric type of flask and 3.9 mL of DPPH containing solution was mixed in a reaction flask and it was placed at room temperature in incubation for about 30 min. Then absorbance was measured at a wavelength of 517 nm using a spectrophotometer. Antioxidant inhibition percentage was estimated with Eq. (3).

3 $${\text{Inhibition}},{\text{ \% }} = \frac{{{\text{Blank }}\;{\text{sample}}\;{\text{absorbance }}{-}{\text{ Sample}}\;{\text{absorbance}}}}{{{\text{Blank}}\;{\text{sample }}\;{\text{absorbance}}}} \times 100$$

Carotenoids were found using a technique recommended by Rodriguez-Amaya ^31^ with certain small changes. For this reason, five grams of vegetable samples were ground, regimented, and taken out in methanol of 10 mL for 2 days. One mL of extract (mg/mL) was added to 18 mL of petroleum ether along with two mL of methanol by making a solution in a proportion of (1:9) using a separating funnel. Two layers were formed in the separating funnel and after the establishment of two layers one layer of solution at the bottom was separated. After decanting the lower layer from the funnel, petroleum ether was introduced into the remaining solution, and the volume was adjusted to 20 mL. This resulting solution was subsequently analysed in a spectrophotometer at a wavelength of 450 nm, using β-carotene as the standard.

The folin-Ciocalteu procedure anticipated by Ismail et al.^32^ was taken as standard procedure to calculate phenolic content with few variations. Five grams of vegetables were ground, homogenized, and taken out in methanol of 10 mL for 2 days. One mL (mg/mL) of extracts of vegetable samples was gathered independently with one mL 1N F–C and 4.6 mL distilled water. After 3 min 3 mL of 2% NaCO_3_ was added to the solution and left for almost 2 h. Then the solution was analysed at a wavelength of 760 nm using a spectrophotometer.

Flavonoids in vegetable samples were determined with the technique anticipated by Ismail et al.^32^. Five grams of vegetable samples were ground and put in 10 mL of methanol for extraction of phytochemicals for 2 days. Then one mL of sample (mg/mL) was taken from diverse samples and four mL of distilled water, 0.3 mL of 10% aluminium chloride, and 2 mL of one molar sodium hydroxide in a flask, and volume was made up to 10 mL with distilled water. Then at 510 nm absorbance of samples was checked using a standard curve of Quercitine using a spectrophotometer.

### Statistical analysis

Statistical procedure Proc GLM program of SAS software (version 9.0) was used to statistically analyse the data obtained from laboratory analysis of vegetable samples^33^. The data were subjected to ANOVA for the level of significance (≤ 0.05) of difference and to Fisher protected Least Significant Difference (LSD) test for differentiation of mean values. Statistical results were presented in the form of bar charts and tables.

### IUCN policy statement

The collection of plant material complies with relevant institutional, national, and international guidelines and legislation.

### Plant collection approval

No approval is needed to collect *Brassica juncea* in Pakistan for research purposes.

## Data Availability

All data generated or analyzed during this study are included in this published article.
