# Author Correction: Physico- and phytochemical properties of *Brassica juncea* as affected by agroclimatic conditions

**DOI:** 10.1038/s41598-024-52693-1

**Published:** 2024-01-25

**Authors:** Uzma Batool, Rab Nawaz, Sajjad Ahmad, Muhammad Atif Irshad, Ali Irfan, Abdel‑Rhman Z. Gaafar, Muhammad Arshad, Gezahign Fentahun Wondmie, Mir Muhammad Nasir Qayyum, Mohammed Bourhia

**Affiliations:** 1 State Key Laboratory of Food Science and Technology, Jiangnan University, Wuxi, Jiangsu 214122 China; 2 Department of Environmental Sciences, The University of Lahore, Lahore, 54000 Pakistan; 3 Faculty of Engineering and Quantity Surveying, INTI International University, 71800 Nilai, Negeri Sembilan Malaysia; 4 Department of Civil Engineering, COMSATS University Islamabad, Sahiwal Campus, Sahiwal, 57000 Pakistan; 5 Department of Chemistry, Government College University Faisalabad, Faisalabad, 38000 Pakistan; 6 Department of Botany and Microbiology, College of Science, King Saud University, P.O. Box 11451, Riyadh, Saudi Arabia; 7 Department of Biology, Bahir Dar University, P.O. Box 79, Bahir Dar, Ethiopia; 8 Department of Chemistry and Biochemistry, Faculty of Medicine and Pharmacy, Ibn Zohr University, Laayoune, Morocco; 9 Laboratory of Chemistry‑Biochemistry, Environment, Nutrition, and Health, Faculty of Medicine and Pharmacy, University Hassan II, B. P. 5696, Casablanca, Morocco; 10 Department of Agriculture & Food Technology, Karakoram International University, Gilgit, 15100 Pakistan

Correction to: *Scientific Reports* 10.1038/s41598-023-48808-9, published online 08 January 2024

In the original version of this Article Muhammad Arshad and Mir Muhammad Nasir Qayyum were incorrectly affiliated with ‘State Key Laboratory of Food Science and Technology, Jiangnan University, Wuxi, Jiangsu, 214122, China’. The correct affiliation is listed below.

Department of Agriculture & Food Technology, Karakoram International University, Gilgit 15100, Pakistan.

The original Article has been corrected.

